# Supplementary material for: Patterns of Intron Gain and Loss in Fungi
Source: PLoS Biol. 2004 Nov 30;2(12):e422. doi: 10.1371/journal.pbio.0020422 (PMC532390; doi:10.1371/journal.pbio.0020422)
Supplement: Table S1 — Also available at http://genes.mit.edu/NielsenEtAl/. (4.3 MB ZIP). [file pbio.0020422.st001.zip › NielsenEtAl/html/1393.html]

AN8023.1.NCU04100.1.MG09517.1.FG07172.1


```
 CLUSTAL W (1.82) Multiple Sequence Alignments - Introns Inserted


Sequence 1: NCU04100.1	706 aa
Sequence 2: FG07172.1	695 aa
Sequence 3: MG09517.1	698 aa
Sequence 4: AN8023.1	696 aa
Alignment Length: 707 aa
Number Identitical Residues: 500 aa
Alignment Score (without introns) 20559


MG09517.1 	---MAAQTSLSTP~GGISDPALIT2LVNKLQDVFATVG~VTNPIDLPQIVVVGSQSSGKS
NCU04100.1	MTVDVNTGSLSTP~GGISDPGLIK2LVNKLQDVFTTVG~VNNPIDLPQIVVVGSQSSGKS
FG07172.1 	-----MSGSLVAQ~GGISDPALIT2LVNKLQDVFATVG~VNNPIDLPQIAVVGSQSSGKS
AN8023.1  	----MASPTTGYS2INVNDPSLIS~LVNKLQDVFSTVG0VQNPIDLPQIAVVGSQSSGKS
          	        :      .:.**.**. *********:*** * ********.**********

MG09517.1 	SVLENIVGRDF2LPRGSGIVTRRPLVLQLHNRPASQSNGVNEEIAGGTDKHANADEWGEF
NCU04100.1	SVLENIVGRDF2LPRGSGIVTRRPLVLQLINRTATQN-GFGNELDDNTDKAANTDEWGEF
FG07172.1 	SVLENIVGRDF2LPRGSGIVTRRPLVLQLINRPAESNSASAEEIDTSNDKQANADEWGEF
AN8023.1  	SVLENIVGRDF2LPRGSGIVTRRPLILQLINKPSQTN-GVKDEQLETTDKEANLDEYGEF
          	*********** *************:*** *:.:  . .  :*    .** ** **:***

MG09517.1 	LHITGQKFYDFNKIRDEITRETEAKVGRNAGISPQPINLRIYSPNVLTLTLVDLPGLTKV
NCU04100.1	LHIPGQKFYDFNKIRDEISRETEAKVGRNAGISPAPINLRIYSPNVLNLTLVDLPGLTRV
FG07172.1 	LHAPGQKFYDFSKIRDEISRETEAKVGKNAGISPAPINLRIYSPNVLTLTLVDLPGLTKV
AN8023.1  	LHIPGQKFYDFNKIRDEIVRETETKVGRNAGISAAPINLRIYSPNVLTLTLVDLPGLTKV
          	** .*******.****** ****:***:*****. ************.**********:*

MG09517.1 	PVGDQPRDIEKQIREMVMKYISKPNAIILAVTPANSDLANSDGLKMAREVDPEGQRTIGV
NCU04100.1	PVGDQPRDIEKQIRDMILKYIQKSNAIILAVTAANVDLANSDGLKLAREVDPEGQRTIGV
FG07172.1 	PVGDQPRDIERQIREMVLKHIGKSNAIILAVTAANQDLANSDGLKLAREVDPEGQRTIGV
AN8023.1  	PVGDQPKDIERQIRDMVLKYISKPNAIVLAVTAANQDLANSDGLKLAREVDPEGQRTIGV
          	******:***:***:*::*:* *.***:****.** *********:**************

MG09517.1 	LTKVDLMDEGTDVVDILAGRIIPLRLGYVPVVNRGQRDIDNKKPIQAALENEKNFFDNHK
NCU04100.1	LTKVDLMDEGTDVVDILAGRIIPLRLGYVPVVNRGQRDIDNKKPINASLEAEKNFFENHK
FG07172.1 	LTKVDLMDEGTDVIDILSNRVIPLRLGYVPVVNRGQRDIDNRKAINQALEAEKNFFENHK
AN8023.1  	LTKVDLMDEGTDVVDILAGRIIPLRLGYVPVVNRGQRDIENKRPISYALEHEKNFFESHK
          	*************:***:.*:******************:*::.*. :** *****:.**

MG09517.1 	AYRNKSSYCGTPYLARKLNL~ILMMHIKQTLPDIKARISSSLQKYSAELESLGPSILGDS
NCU04100.1	AYRNKSSYCGTPYLARKLNL~ILMMHIKQTLPDIKARISSSLQKYTQELESLGPSILGNS
FG07172.1 	AYRNKSSYCGTPYLARKLNL~ILMMHIKQTLPDIKARISSSLQKYTAELESLGPSMLGNS
AN8023.1  	AYRNKASYCGTPYLARKLNL0ILMMHIKQTLPDIKSRISSSLQKYSSELAQLGDSMLGNS
          	*****:************** **************:*********: ** .** *:**:*

MG09517.1 	TNIVLKVITDFSGEWKDVLDGNNTEISGQELSGGARISFVFHELYANGIKAVDPFDVVKD
NCU04100.1	ANIVLNIITEFTNEWRTVLDGKSTELSSQELSGGARISFVFHELYSNGVKAVDPFDQVKD
FG07172.1 	ANIVLNIITEFTNEWRTVLDGNNTELSSTELSGGARISFVFHELYSNGVKAIDPFDVVKD
AN8023.1  	ANIILNIITEFSNEYRTVLEGNNQELSSVELSGGARISFVFHELYSNGIKAVDPFDQVKD
          	:**:*::**:*:.*:: **:*:. *:*. ****************:**:**:**** ***

MG09517.1 	QEIRTFIYNSSGPSPALFVGTGAFEQIVKKQIARLEEPSLKCISLVYDELVRILSQLLAK
NCU04100.1	SDIRVILYNSSGPSPALFVGTAAFELIVKQQIKRLEEPSLKCASLVYDELVRILTTVLSK
FG07172.1 	VDIRTILYNSSGSSPALFVGTTAFELIVKQQIKRLEDPSLKCVSLVYDELVRILSQLLAK
AN8023.1  	IDIRTILFNSSGSSPALFIGTTAFELIVKQQIARLEDPSLKCISLVYDELVRILGQLLNK
          	 :**.:::****.*****:** *** ***:** ***:***** ***********  :* *

MG09517.1 	QLYRRYPQLKEKFHQVVVAFFKKAMEPSVKLVKDLVAMEVCYINTGHPDFLNGHR0AMAI
NCU04100.1	QLYRRYPGLKEKIHQVVISFFKKAMEPTNKLVKDLVAMEACYINTGHPDFLNGHR~AMAI
FG07172.1 	QLYRRYPSLKEKMHGVVIAFFKKAMEPTNKLVRDLVSMESCYVNTGHPDFLNGHR0AMAM
AN8023.1  	QLFRRYPMLKEKFHAVVIAFFKKAMEPTNKLVRDLIAMETTYINTAHPDFLNGHR0AMAI
          	**:**** ****:* **::********: ***:**::**  *:**.********* ***:

MG09517.1 	VNERMNPKPVTQVDPKTGKAIST--TPARTASPGVPDS----GDGNSGFFGSFFAGKNKK
NCU04100.1	VNERHNGSRPVQVDPKTGKPIPQSATPARAASPTLAGSNLDEGGSNGGFFGSFFAAKNKK
FG07172.1 	VNERYN-PKPVQVDPKTGKPLAG---TPRAASPTVPDG---ENAGNSGFFGSFFAAKNKK
AN8023.1  	VNERHAAAKPTQVDPKTGKPLPP-----RANSPSVEATTP-DTNSSSGFFGSFWASKNKK
          	****      .********.:.      *: ** :   .  .  ...******:*.****

MG09517.1 	KAAAMEPPPPMLKASGNLSEREAMEVEVIK1LLIASYFNIVKRTMIDMVPKAIMLNLVQ2
NCU04100.1	KAAAMEPPPPSLKASGTLSERENIEVEVIK~LLIQSYYNIVKRTMIDMVPKAIMLNLVQ2
FG07172.1 	KAAAMEAPPPTLKASGTLSERENIEVEVIK1LLISSYYNIVKRTMIDMVPKAVMLNLVQ2
AN8023.1  	KMAAMEAPPPNLKASAALSERESTEVEVIK1LLITSYFNIVKRTMIDMVPKAIMYTLVQ2
          	* ****.*** ****. *****  ****** *** **:**************:* .*** 

MG09517.1 	YTKENMHAELLASMYKNEQLDDLLKESDYTIRRRKECQQMVESLSRAQEIVGQVQ
NCU04100.1	LTKDEMQKELLENMYRADELDDLLKESDYTVRRRKECQQMVESLSKASEIVSQVQ
FG07172.1 	FTKDEMQRELLENMYRTDTLDDLLKESDFTIRRRKECQQMVESLGKASEIVSQVQ
AN8023.1  	YSKEGMQRELLENMYRNAELDDLLKESDYTIRRRKECQQMVESLSRASEIVSQVQ
          	 :*: *: *** .**:   *********:*:*************.:*.***.***
```
